# Preparation and Characterization of Regenerated Cellulose Film from a Solution in Lithium Bromide Molten Salt Hydrate

**DOI:** 10.3390/polym10060614

**Published:** 2018-06-04

**Authors:** Xueqin Zhang, Naiyu Xiao, Huihui Wang, Chuanfu Liu, Xuejun Pan

**Affiliations:** 1 College of Light Industry and Food, Zhongkai University of Agriculture and Engineering, Guangzhou 510225, China; xueqin0228@gmail.com; 2 State Key Laboratory of Pulp and Paper Engineering, South China University of Technology, Guangzhou 510640, China; wang.huihui@mail.scut.edu.cn; 3 Department of Biological System Engineering, University of Wisconsin-Madison, 460 Henry Mall, Madison, WI 53706, USA; xpan@wisc.edu

**Keywords:** inorganic ionic liquid, kraft pulp, non-derivatizing dissolution, packaging film

## Abstract

In this study, the molten salt hydrate of lithium bromide (LiBr) was utilized as a non-derivatizing cellulose dissolution solvent to prepare regenerated cellulose films for kraft pulp. The effects of LiBr concentrations (60, 62, and 65 wt %) and dissolving time (from 5 to 40 min with the interval of 5 min) on the structures and the properties of the films were investigated. Fourier transform infrared (FT-IR) and cross-polarization magic-angle spinning carbon-13 nuclear magnetic resonance (CP/MAS ^13^C NMR) characterizations verified the breakage of inter- and intra-cellulose hydrogen bonds during the regeneration, resulting in the disruption of the crystalline structure of cellulose. X-ray diffraction (XRD) data indicated that the regeneration converted the polymorphism of cellulose from I to II as well as decreased its crystallinity. Ultraviolet-visible spectra (UV-Vis) and scanning electron microscopy (SEM) analyses revealed the excellent optical transparency of the films to visible light due to the complete dissolution of cellulose fibers as well as the sufficient breaking of the inter- and intra-cellulose hydrogen bonds. In terms of tensile testing, tuning LiBr concentrations and dissolving time could increase the elongation at break and tensile strength of the films. The maximum elongation at break of 26% and tensile strength of 67 MPa were achieved when the films prepared in 65 wt % LiBr for 10 and 15 min, respectively. These results indicated the great potential of the cellulose films for packaging use.

## 1. Introduction

With the urgent demand to address the energy and environment issues that are caused by using fossil resources, there have been increasing interests in exploring biomass-based renewable and sustainable materials. Cellulose, a linear polymer of glucose with linkage of *β*-1,4-glycosidic bond, is the most abundant polysaccharide on earth, and has been utilized for various types of biocomposites [1]. Among which, cellulose-based films have drawn considerable attention for packaging, including food packaging [2,3], because of their optical transparency, tunable porous structure, and good mechanical properties [4]. However, the insolubility of natural cellulose in water and common organic solvents due to the highly crystalline structure makes it very challenging to prepare the films directly from celluloses [5]. 

In recent years, many solvent systems have been developed to negate the dissolution recalcitrance of cellulose to prepare regenerated films [4]. Generally, the solvent systems can be divided into derivatizing and non-derivatizing ones [6]. Non-derivatizing solvent systems have been extensively studied for dissolving and regenerating cellulose, as well as developing advanced and homogeneous cellulose derivatives [6]. A well-known cellulose non-derivatizing solvent system is *N,N*-dimethylacetamide (DMAc) or its cyclic form, 1-methyl-2-pyrrolidinone (NMP) with lithium chloride (LiCl) [7,8]. It was reported that this solvent with the concentration of LiCl 1–10 wt % was able to dissolve cellulose up to 10–15 wt % [9]. Ionic liquids, firstly proposed by Rogers in 2002, is another cellulose non-derivatizing solvent system [10]. So far, ionic liquids have been comprehensively studied in the areas of dissolving and derivatizing lignocelluloses [11,12,13]. Compared to 10 wt % DMAc/LiCl, an ionic liquid, such as 1-buthyl-3-methylimidazolium chloride ([C_4_mim]Cl), had almost three times more free chloride ion concentration, which played a crucial role in breaking the hydrogen-bonding network in cellulose [10]. The third well-known solvent system is NaOH- or LiOH-urea aqueous system working at low-temperature, which was developed by Zhang’s research group [14,15]. Additional examples of non-derivatizing solvents include *N*-methyl-*N*-morpholine-*N*-oxide (NMMO)/water [16], concentrated phosphoric acid [17], and aqueous NaOH solutions [18]. However, these solvents still more or less suffered from different drawbacks, such as the environmental problems, complicated multistep processes, or high price [19,20]. 

Molten salt hydrates have been recently found to be as an economic, recyclable, and non-toxic non-derivatizing solvent system for cellulose [21]. Unlike many non-derivatizing solvents, cellulose dissolution in molten salt hydrates did not require pretreatment, which makes molten salt hydrate an attractive cellulose solvent [22]. In a molten salt hydrate, the water-salt molar ratio is close to the coordination number of the strongest hydrated ion, normally a cation [23]. The molten salt hydrates reported to be able to dissolve cellulose include ZnCl_2_·4H_2_O, LiI·2H_2_O, LiSCN·2H_2_O, as well as a eutectic mixture of NaSCN/KCN/LiSCN·3H_2_O [22,24]. The dissolving capacity of molten salt hydrates mainly depends on the species of the molten salts, the state of the solution, and the water content. 

The molten salt of hydrate lithium bromide (concentrated aqueous solution of LiBr), which is usually used as liquid desiccants for its low water vapor pressure [25], was recently found to be able to dissolve cellulose and was used in the regeneration, functionalization, and hydrolysis of cellulose [17,26,27]. It was observed that the cellulose-LiBr solution could easily form a gel when the solution was cooled down to approximately 70 °C. Cellulose biocomposites could be then easily fabricated after removing the salts via water washing [17]. 

In this study, cellulose films from kraft pulp were prepared by direct dissolution and the regeneration of cellulose in LiBr molten salt hydrate. The effects of LiBr concentrations and dissolving time on the structures and properties of the resultant cellulose films were investigated. From our preliminary experiments, the results (not shown here) indicated that cellulose could not be dissolved in LiBr solution with concentrations that were less than 60 wt % within 1 h. In addition, when the concentration of LiBr solution is higher than 65 wt %, the LiBr solids will be precipitated at room temperature. Therefore, the LiBr molten salt hydrates with the concentrations of 60 wt %, 62 wt %, and 65 wt % were used in this study. The regenerated cellulose films were thoroughly characterized by using Fourier transform infrared (FT-IR), cross-polarization magic-angle spinning carbon-13 nuclear magnetic resonance (CP/MAS ^13^C NMR), Ultraviolet-visible spectra (UV-Vis), scanning electron microscopy (SEM), and X-ray diffraction (XRD) analyses. The optical and mechanical strength of the films were evaluated as well. 

## 2. Materials and Methods

### 2.1. Materials

A bleached hardwood kraft pulp was used as cellulose source. The pulp board was shredded and soaked in distillated water at room temperature for 24 h. Then, it was mechanically disintegrated and freeze dried for later use. The average degree of polymerization (DP) of cellulose was 768, determined using viscosity method following the GB/T 1548-2016 standard method. 

LiBr (99%) was purchased from Macklin Reagent Co. (Shanghai, China). Aqueous LiBr solutions with various concentrations (60, 62, and 65 wt %) were prepared by dissolving LiBr in water.

### 2.2. Cellulose Film Preparation in Aqueous Libr Solutions

A typical process for preparing cellulose film was as follows. First, 0.2 g cellulose was stirred in 15 g LiBr molten salt hydrate solution at room temperature for about 3 h to ensure the full swelling of the cellulose. Then, the mixture was heated up to 130 °C to dissolve the cellulose. After that, the solution was quickly cast onto a glass plate to get a hydrogel, which was immediately immersed into deionized water to completely remove LiBr (confirmed by the AgNO_3_ test). The obtained transparent gel was pressed between two filter papers and then dried naturally in air to get a cellulose film. Films were named as x-y, according to their fabrication conditions. The x stands for the concentration of LiBr (%) and y is the dissolution time (min). For example, sample 65%-5 represents the film that was prepared in 65 wt % LiBr with dissolution time of 5 min. 

### 2.3. Characterization

Fourier transform infrared (FT-IR) spectra were obtained in the range of 4000–500 cm^−1^ on a Tensor 27 spectrophotometer (Bruker, Karlsruhe, Germany) in KBr disc containing 1% (*w*/*w*) of finely ground sample. Thirty-two scans were applied with 4 cm^−1^ resolution in the transmittance mode.

Solid-state cross-polarization magic-angle spinning carbon-13 nuclear magnetic resonance (CP/MAS ^13^C NMR) analysis was performed on a Bruker AVANCE III 600 spectrometer (Karlsruhe, Germany) at a resonance frequency of 150.9 MHz. The MAS probe of 4 mm and the spinning rate of 12 kHz were used. The contact time was 3 ms and the recycle delay was 3 s. The chemical shifts of ^13^C were externally referenced to TMS.

Ultraviolet-visible spectra (UV-Vis) were recorded on a TU-1810 spectrophotometer (Beijing, China) within the scan range of 200 to 900 nm.

The surface morphology of films was observed by using scanning electron microscopy (SEM) on a LEO 1530 VP equipment (Oberkochen, Germany) with an accelerating voltage of 10 kV. Cellulose film sample was attached onto a mica sheet followed by gold-plating for SEM observation.

The tensile strength of the prepared films was tested using rectangular specimens (15 mm × 10 mm) on an Instron Universal Testing Machine 5565 (Instron, Norwood, MA, USA) with a 100 N load cell at 23 °C and 50% RH. Five replicate specimens were tested for each film sample. The initial distance between the two grips was 30 mm and the separation rate of the grips was 4 mm/min.

The crystallinity of samples was characterized by X-ray diffraction (XRD) measurement on a D/max-III X-ray diffractometer (Rigaku, Tokyo, Japan), equipped with nickel-filtered Cu Kα radiation (λ = 0.15418). The diffraction angle (2*θ*) ranged from 5° to 40° and the step size was 0.04°. Lorentz deconvolution was used for peak separations. The *d*-spacings (*d*) of films were calculated with the Bragg equation [28]:

(1) $$d=\frac{\lambda }{2\sin\theta }$$

where *λ* is the wavelength of the X-ray source (0.15418 nm) and *θ* is the Bragg angle corresponding to the plane.

The apparent crystallite size (*L*) of the reflection plane was obtained using the Scherrer equation [29]:

(2) $$L=\frac{K\lambda }{\beta \cos\theta }$$

where *K* is the Scherrer constant of 0.94 and *β* is the half-height width of the diffraction band.

The surface chains of cellulose crystals occupy a layer approximately 0.57 nm thick (*h*); therefore, the proportion of crystallite interior chains (*X*) was calculated with the following equation [30]:

(3) $$X={\left(\frac{L-2h}{L}\right)}^{2}$$

The Segal crystalline index (CrI) [31] was calculated, as follows:

(4) $$\mathrm{CrI}=\frac{I_{200}-I_{am}}{I_{200}}$$

where *I*_200_ is the intensity of the peak at 2*θ* ≈ 22° corresponding to (200) and *I_am_* is the amorphous intensity at 2*θ* ≈ 18° for cellulose I and 2*θ* ≈ 16° for cellulose II.

## 3. Results and Discussion

### 3.1. FT-IR Analysis

**Figure 1:**
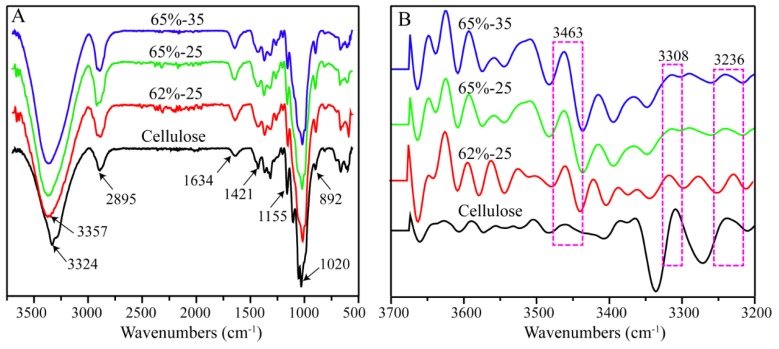
Fourier transform infrared (FT-IR) spectra (**A**) and relative second-derivative FT-IR spectra (**B**) of cellulose samples.

FT-IR was used to investigate the changes in chemical structure of cellulose during the dissolution and regeneration in LiBr molten salt hydrate. As shown in Figure 1A, the main functional groups in cellulose were detected at the wavenumbers of 3324 (O-H hydroxyl group stretching vibration), 2895 (–CH_2_– alkyl stretching vibration), 1421 (–CH_2_– alkyl bending stretching), 1155 (C–O–C pyranose ring skeletal vibrations), 1020 (C–O stretching) and 892 cm^−1^ (*β*-glucosidic linkages between the sugar units), respectively. The absorption at 1634 cm^−1^ is attributed to the absorbed water. The same characteristic bands of cellulose were observed on the spectra of the regenerated films (62%-25, 65%-25, and 65%-35), indicating that cellulose structure did not change during the dissolution and regeneration. This observation verified the non-derivatizing nature of LiBr molten salt hydrate as cellulose solvent. However, the O-H stretching vibration shifted to a higher wavenumber domain of 3357 cm^−1^, which was probably due to the decreased hydrogen bonding in cellulose after regeneration [32]. Moreover, the intensity of the weak absorption at 892 cm^−1^ attributed to the C–H deformation at anomeric center of *β*-glucosidic linkages increased, which was in agreement with the previous results [17]. 

The second-derivatives of FT-IR spectra were conducted to further confirm the changes of hydrogen bonding of cellulose upon regeneration. Apparently, the intensity of peaks at 3236 (the interchain O2-H^…^O6 bonding of I*_β_*) and 3308 cm^−1^ (the O2-H^…^O6-H^…^O3-H^…^O5 bonding) decreased, while that of the band at 3463 cm^−1^ increased (the OH groups with weak hydrogen bonding in the semicrystalline domains [33]). These results indicated that the dissolution and the regeneration of cellulose in LiBr molten salt hydrate weakened or broke the inter- and intra-cellulose hydrogen bonding, and thereby disrupted its crystalline structure. In addition, the effects increased with the severity of the treatment conditions. 

### 3.2. CP/MAS ^13^C NMR Analysis

**Figure 2:**
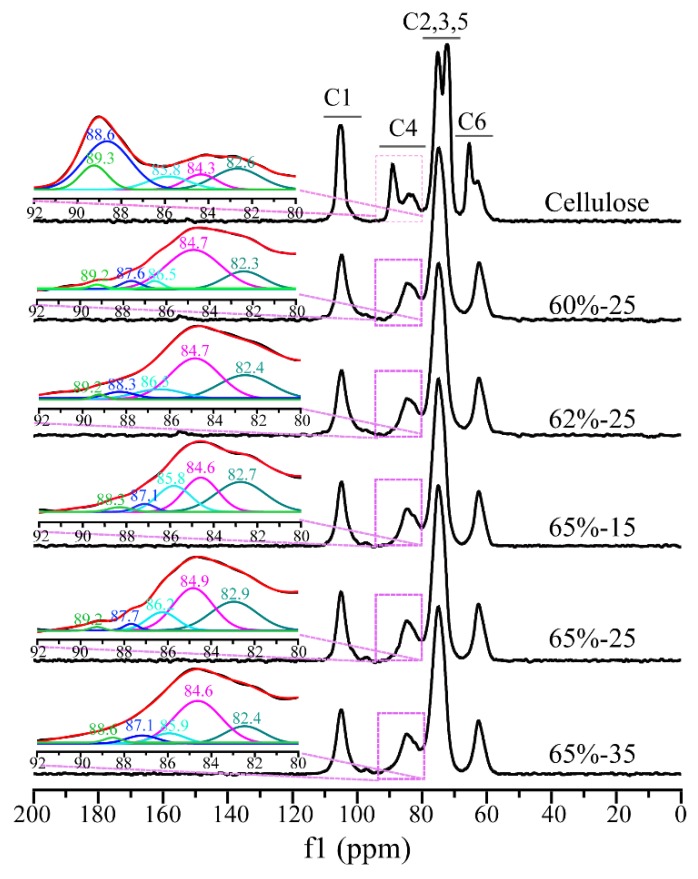
Cross-polarization magic-angle spinning carbon-13 nuclear magnetic resonance (CP/MAS ^13^C NMR) spectra of cellulose samples.

The CP/MAS ^13^C NMR spectra of cellulose and regenerated cellulose films are shown in Figure 2. The ^13^C NMR spectrum of native cellulose has four signal clusters: the region at 102–108 ppm assigned to C1 moieties; the two signals at the region of 79–92 ppm assignable to C4 moieties due to amorphous (83 ppm) and crystalline (89 ppm) states, respectively; a cluster of signals from C2, C3, and C5 carbons (68–79 ppm); and, that in the region at 57–68 ppm including a broad signal at 63 ppm and a sharp signal at 65 ppm assignable to C6 moieties for the amorphous and crystalline states, respectively. For the regenerated cellulose films, the structures of the glucose rings of cellulose retained intact, verifying again the non-derivatizing dissolution of cellulose in LiBr molten salt hydrate. However, the regenerated films had overlapping signals of C2, C3, and C5 (68–79 ppm), accompanied by the disappearance of the signals at 89 and 65 ppm for the crystalline structure at C4 and C6, as well as the decreased intensity of the amorphous signals at C4 (83 ppm) and C6 (63 ppm), respectively, indicating the disruption of the crystalline structure of cellulose after dissolution and regeneration in LiBr molten salt hydrate [17]. 

In general, when cellulose crystallinity is lower than 50%, it is not reliable to compare the difference of cellulose polymorphs using C6 resonance [34]. Alternatively, C4 signal could be used [35]. Therefore, the Gaussian deconvolution of C4 signal was adopted to investigate the polymorphs difference of cellulose during the dissolution and regeneration, as shown in Figure 2. When compared to the original cellulose, the intensity of the signals at 89.3 and 88.6 ppm for cellulose *I_α_* and cellulose *I_β_*, respectively, of regenerated cellulose dramatically decreased, while that of the signal at 84.6 ppm attributing to accessible crystallite surfaces increased. These results further confirmed the disruption of crystallite structure of cellulose during the dissolution and regeneration in LiBr molten salt hydrate, which is consistent with the FT-IR data above. 

### 3.3. XRD Analysis

**Figure 3:**
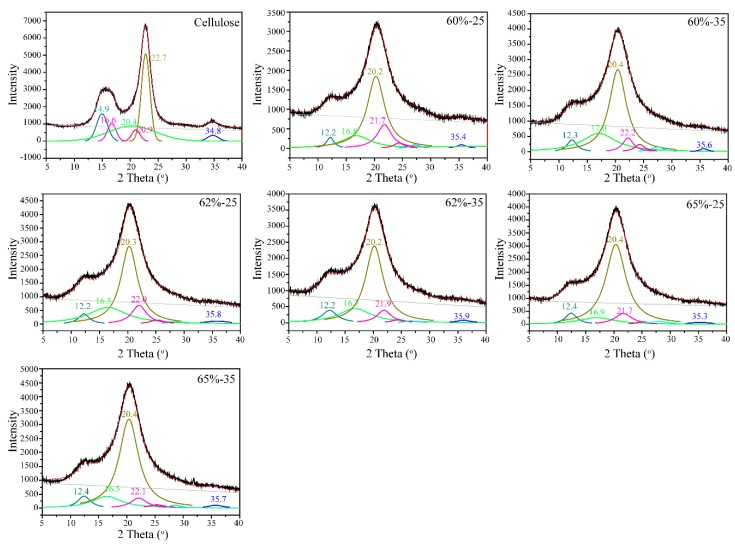
Lorentz deconvolution of the X-ray diffraction (XRD) patterns of cellulose samples.

XRD was carried out to compare the crystallinity of cellulose before and after the regeneration, as shown in Figure 3. The original cellulose displayed a typical cellulose I XRD pattern with the specific peaks, as is consistent with the literature [36]. The regenerated films presented typical cellulose II characteristics with peaks at about 2*θ* = 12.2° and 20.4°, associating to (110) and ($\bar{1}$10) planes, respectively, indicating the transformation of the crystal structure from cellulose I to cellulose II upon the dissolution and regeneration [23]. The Lorentz deconvolution process was applied to distinguish amorphous and crystalline contributions to the diffraction spectrum. The related parameters, including *d*-spacings (*d*), the crystallite size (*L*), the proportion of crystallite interior chains (*X*), and the crystallinity index (CrI) were calculated from XRD profiles and are summarized in Table 1 and Table 2. 

For native cellulose, Miller indices of ($\bar{1}$10), (110), (200), and (004) illustrated the orientation of the original cellulose crystallites along the fiber axis [37]. After the dissolution and regeneration in LiBr molten salt hydrate, the appearance of the peaks in the 2*θ* range of 25° to 35° upon the Lorentz deconvolution suggested the random orientation of the cellulose crystallites. These observations indicated that the dissolution and regeneration in LiBr molten salt hydrates could disrupt the cellulose crystalline structures [37]. As shown in Table 1, the *d*-spacings of 0.595 ($\bar{1}$10), 0.534 (110), 0.392 (200), and 0.258 nm (004) are attributed to the cellulose I*_β_* crystalline stacks for native cellulose, respectively. Differently, the regenerated cellulose film from LiBr molten salt hydrate showed no discernible impact on the (110), (200), and (004) planes, but an apparent increase on the ($\bar{1}$10) plane, further indicating the transformation of cellulose I to cellulose II during the dissolution and regeneration. Additionally, the *L*, *X,* as well as the CrI of regenerated cellulose all considerably decreased, indicating that regeneration in LiBr molten salt hydrate decreased the crystallinity of cellulose and increased the exposure of the cellulose chains, which is in agreement with the ^13^C NMR data above. 

### 3.4. SEM Analysis

**Figure 4:**
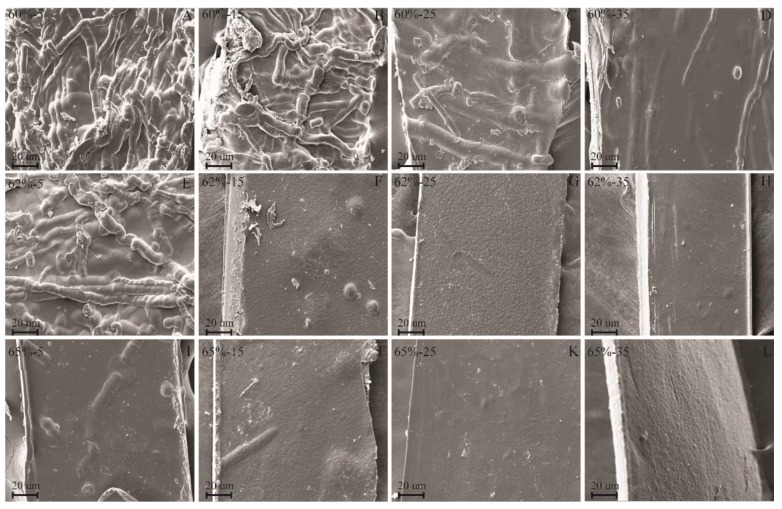
Scanning electron microscopy (SEM) images of the regenerated cellulose films ((**A**) for 60%-5, (**B**) for 60%-15, (**C**) for 60%-25, (**D**) for 60%-35, (**E**) for 62%-5, (**F**) for 62%-15, (**G**) for 62%-25, (**H**) for 62%-35, (**I**) for 65%-5, (**J**) for 65%-15, (**K**) for 65%-25 and (**L**) for 65%-35).

In order to investigate the effects of the LiBr concentrations and dissolving time on the surface morphology of the regenerated films, SEM analysis was conducted, as shown in Figure 4. From Figure 4A, cellulose fibers were still clearly visible in the film that was prepared in 60 wt % LiBr molten salt hydrate for 5 min. This result indicated that low LiBr concentration and short dissolving time were insufficient to completely dissolve cellulose [36]. With the extension of dissolving time from 5 min to 15 min (B), and then to 25 min (C), the cellulose fibers remarkably reduced in the films, and finally became almost invisible at 35 min (D). These results indicated that extending dissolving time in LiBr molten salt hydrate with low concentration could increase the dissolving of cellulose fibers, but not totally dissolve. Increasing the LiBr concentration to 62 wt % resulted in the significant decrease of the cellulose fibers after 5 min (E), and then disappeared after 15 min (F). For the films that were regenerated in 65 wt % LiBr, the cellulose fibers were almost invisible after 5 min (I). These are probably due to the destruction of the cellulose crystalline structure and the increased dissolution of cellulose with the development of the LiBr concentration and dissolution time. However, small nodules and contours were observed in the films (F–L), which resulted from the formation of strong hydrogen bonding of cellulose during film-forming [38,39].

### 3.5. UV-Vis Spectrophotometric Analysis

**Figure 5:**
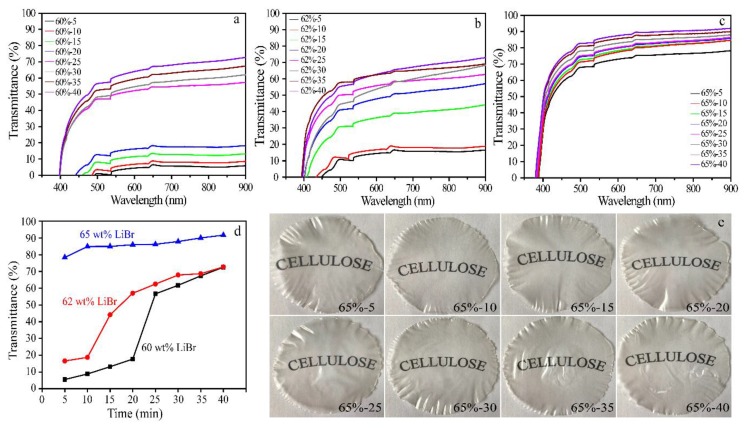
Light transmittance graphs of cellulose films prepared under various conditions (**a**–**c**), and plotting the light transmittance of cellulose films (**d**), and the photographs of cellulose films prepared in 65 wt % LiBr molten salt hydrate with varied dissolution time (**e**).

The transparency of the films prepared in LiBr solutions with various concentrations and dissolving time was evaluated using UV-Vis spectrophotometer, as shown in Figure 5. In the visible region (400–800 nm), the light transmittance of films was improved with the increment of LiBr concentration and the extension of dissolving time (Figure 5d). For the films prepared in 60 wt % and 62 wt % LiBr solution, the maximum light transmittances of films were only 72% and 73%, respectively (Figure 5a,b). Comparatively, the light transmittance of the films regenerated in 65 wt % LiBr solution was above 80% (Figure 5c). Similar results could be observed on the cellulose films regenerated in dimethylacetamide/lithium chloride (DMAc/LiCl), 1-ally-3-methylimidazolium ([Amim]Cl), and LiOH/urea/H_2_O with the extension of dissolving time [17,18,36]. Figure 5 also lists the photographs of the transparency of the films that were prepared in 65 wt % LiBr with different dissolving time. As shown in Figure 5e, the longer the dissolving time was, the higher the optical transparent of the films became. These are probably due to the decrease of the crystallinity of cellulose and the complete dissolution of cellulose with the increase of the LiBr concentration and the extension of dissolving time. However, the surface of the film 65%-40 became damaged, which is probably due to the dramatic disruption of the crystalline structure of cellulose under severe dissolving conditions. Although SEM images showed the disappearance of cellulose fibers in 62 wt % LiBr solution after 15 min, the light transmittances of resultant films were still lower than 80%, suggesting that cellulose was not completely dissolved in low-concentrations (<65 wt %) LiBr solutions.

### 3.6. Mechanical Properties of Films

**Figure 6:**
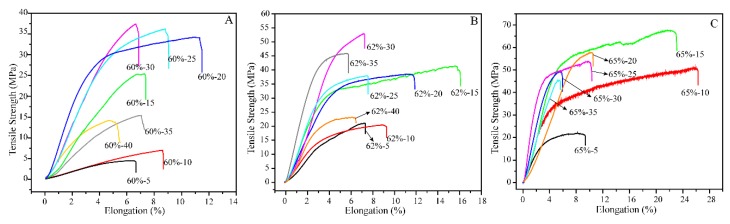
Tensile strain curves of cellulose films regenerated in 60 wt % (**A**), 62 wt % (**B**) and 65 wt % (**C**) LiBr molten salt hydrate with different dissolving time.

Figure 6 shows the tensile stress-strain curves of the regenerated cellulose films prepared in 60, 62 and 65 wt % LiBr molten salt hydrates with different dissolving time, respectively, and the corresponding tensile strength, Young’s modulus, and elongation at break of films are summarized in Table 3. Apparently, in 60 wt % LiBr molten salt hydrate (Figure 6A), extending the dissolving time from 5 to 20 min resulted in an increment of the tensile strength and elongation at the break of the cellulose films from approximately 4 MPa and 7% to 34 MPa and 12%, respectively. These results are probably due to the increased dissolution of cellulose. Further extending the dissolving time from 20 to 30 min, and to 40 min led to an increase of the tensile strength of films from approximately 34 to 38 MPa, and then decreased to approximately 14 MPa, while the elongation at break of films decreased from approximately 12% to 5%. This is probably attributed to the severe disruption of the crystalline structure of cellulose. For the cellulose films that were regenerated in 62 wt % LiBr molten salt hydrate (Figure 6B), the tensile strength and elongation at break of the cellulose films increased from approximately 21 MPa and 7% to 41 MPa and 16%, respectively, when the dissolving time extended from 5 to 15 min. The tensile strength of films reached to the maximum of approximately 53 MPa at 30 min dissolving time, and then decreased with further extension of dissolving time. For example, the tensile strength dropped to approximately 23 MPa at 40 min. Differently, the elongation at break reached the maximum (16%) at 15 min and then declined to 6% with the extension of dissolving time to 40 min. Similar trends were observed for the films prepared in 65 wt % LiBr molten salt hydrate (Figure 6C), but higher tensile strength (67 MPa) and elongation at break (26%) were reached, suggesting that the sufficient and complete dissolution of cellulose in 65 wt % LiBr solution generated not only more homogeneous and transparent (Figure 4 and Figure 5), but also stronger, films. However, when extending the dissolving time to 40 min, the cellulose film (65%-40) was too fragile to test the mechanical properties (as shown in Figure 5e). Comparatively, the tensile strength and Young’s modulus of the cellulose films regenerated in LiBr molten salt hydrate from kraft pulp were lower than those of other regenerated cellulose films [40]. This could probably be attributed to the cellulose obtained from different sources that is used in these studies, as well as the various regeneration conditions.

It is worth mentioning that the films prepared in LiBr molten salt hydrate with appropriate dissolving time had high toughness because of the high ultimate tensile strength and failure strain [41]. Usually, the elongation at break of the cellulose films regenerated in ionic liquid [40], NaOH/urea [42] or DMAc/LiCl [17] was lower than 15%. The films prepared in the present study had the maximum elongation at break as high as 26%. The tensile strength of the cellulose films that were prepared from kraft pulp in LiBr molten salt hydrate was higher than that of widely used commercial polyolefin films, such as polypropylene (PP) and polyethylene (PE) films [43]. With good strength and toughness, in addition to biodegradability, the cellulose films that were prepared in this study have great potential to replace non-biodegradable PP and PE in the packaging area. 

## 4. Conclusions

In the present study, the regenerated cellulose films were prepared from kraft pulp in LiBr molten salt hydrate under different conditions (LiBr concentrations and dissolving time). FT-IR and CP/MAS ^13^C NMR studies indicated that inter- and intra-cellulose hydrogen bonds were broken during the dissolution and regeneration, and as a result, the crystalline structure of cellulose was disrupted. The results of XRD showed that the regeneration in LiBr molten salt hydrate converted the polymorphism of cellulose from I to II, and meanwhile it reduced its crystallinity, which was consistent with the FT-IR and ^13^C NMR results. The good dissolution of cellulose in LiBr molten salt hydrate was confirmed by SEM imaging, and no cellulose fibers were visible when kraft pulp was dissolved in LiBr solution with 62 wt % or above concentration for sufficient dissolving time. The resultant cellulose films had high transparency to visible light. The films that were regenerated in 65 wt % LiBr molten salt hydrate after 10 and 15 min exhibited good toughness with high elongation at break up to 26% and good tensile strength of 67 MPa, respectively. The films are anticipated to have great potential as packaging films.

## Figures and Tables

**Table 1:** The *d*-spacings and the crystalline size of cellulose samples.

Sample	*d*-Spacing (nm)				Crystalline Size (nm)			
	$\bar{1}$10	110	200	004	$\bar{1}$10	110	200	004
Cellulose	0.595	0.534	0.392	0.258	7.35	7.84	8.79	7.47
60%-5	0.738	0.433	0.385	0.258	6.57	4.59	5.58	6.52
60%-15	0.725	0.439	0.411	0.252	4.80	4.49	4.94	4.57
60%-25	0.731	0.437	0.409	0.254	4.64	4.45	4.52	4.47
60%-35	0.720	0.435	0.400	0.252	3.54	4.28	3.94	3.79
62%-5	0.720	0.440	0.410	0.253	6.44	4.54	5.42	6.47
62%-15	0.725	0.442	0.406	0.254	4.69	4.42	4.85	4.47
62%-25	0.725	0.437	0.404	0.251	4.31	4.37	4.38	4.30
62%-35	0.714	0.440	0.406	0.250	3.45	4.20	3.82	3.75
65%-5	0.720	0.439	0.408	0.255	6.38	4.47	5.36	6.31
65%-15	0.708	0.435	0.406	0.254	4.60	4.33	4.67	4.35
65%-25	0.714	0.435	0.409	0.254	4.28	4.29	4.29	4.17
65%-35	0.714	0.435	0.402	0.251	3.28	4.12	3.57	3.37

**Table 2:** The proportion of crystalline interior chains and the crystalline index of cellulose samples.

Sample	Proportion of Crystalline Interior Chains (nm)				CrI
	$\bar{1}$10	110	200	004	(%)
Cellulose	0.713	0.76	0.768	0.718	82.7
60%-5	0.683	0.565	0.628	0.657	75.8
60%-15	0.587	0.558	0.573	0.569	70.3
60%-25	0.576	0.554	0.527	0.548	63.6
60%-35	0.562	0.543	0.490	0.499	60.7
62%-5	0.671	0.558	0.613	0.527	67.9
62%-15	0.582	0.548	0.591	0.427	64.8
62%-25	0.567	0.540	0.546	0.408	62.1
62%-35	0.531	0.528	0.482	0.391	58.4
65%-5	0.628	0.529	0.599	0.497	61.3
65%-15	0.566	0.541	0.559	0.420	59.8
65%-25	0.540	0.538	0.527	0.399	55.8
65%-35	0.519	0.508	0.436	0.383	50.5

**Table 3:** Mechanical properties of cellulose films prepared under different conditions.

Sample	Tensile Strength (MPa)	Young’s Modulus (MPa)	Elongation at Break (%)
60%-5	4.19	343.02	6.68
60%-10	6.74	398.71	8.75
60%-15	25.71	394.33	7.26
60%-20	33.79	727.40	11.60
60%-25	36.80	568.36	8.86
60%-30	37.51	396.06	6.87
60%-35	15.07	355.28	7.16
60%-40	13.93	493.76	5.17
62%-5	20.93	720.58	7.31
62%-10	20.30	573.22	9.23
62%-15	41.22	1210.99	16.09
62%-20	38.32	897.46	11.57
62%-25	37.91	741.68	7.49
62%-30	52.60	964.86	7.27
62%-35	45.77	1277.90	5.71
62%-40	22.79	886.71	6.35
65%-5	20.69	771.60	9.23
65%-10	50.67	771.65	26.03
65%-15	66.80	1506.63	22.96
65%-20	57.59	1362.12	10.43
65%-25	53.75	835.02	9.97
65%-30	49.38	1433.00	5.69
65%-35	42.92	1403.95	5.58
